# The Blood–Brain Barrier and Its Intercellular Junctions in Age-Related Brain Disorders

**DOI:** 10.3390/ijms20215472

**Published:** 2019-11-03

**Authors:** Laura Costea, Ádám Mészáros, Hannelore Bauer, Hans-Christian Bauer, Andreas Traweger, Imola Wilhelm, Attila E. Farkas, István A. Krizbai

**Affiliations:** 1 Institute of Life Sciences, Vasile Goldiş Western University of Arad, 310414 Arad, Romania; laura.m.costea@gmail.com (L.C.); wilhelm.imola@brc.hu (I.W.); 2 Institute of Biophysics, Biological Research Centre, 6726 Szeged, Hungary; meszaros.adam@brc.hu; 3 Doctoral School of Biology, University of Szeged, 6726 Szeged, Hungary; 4 Department of Biological Sciences, University of Salzburg, 5020 Salzburg, Austria; hannelore.bauer@sbg.ac.at; 5 Institute of Tendon and Bone Regeneration, Paracelsus Medical University—Spinal Cord Injury and Tissue Regeneration Center Salzburg, 5020 Salzburg, Austria; hans.bauer@pmu.ac.at (H.-C.B.); andreas.traweger@pmu.ac.at (A.T.); 6 Department of Physiology, Anatomy and Neuroscience, University of Szeged, 6726 Szeged, Hungary

**Keywords:** aging, blood–brain barrier, tight junction

## Abstract

With age, our cognitive skills and abilities decline. Maybe starting as an annoyance, this decline can become a major impediment to normal daily life. Recent research shows that the neurodegenerative disorders responsible for age associated cognitive dysfunction are mechanistically linked to the state of the microvasculature in the brain. When the microvasculature does not function properly, ischemia, hypoxia, oxidative stress and related pathologic processes ensue, further damaging vascular and neural function. One of the most important and specialized functions of the brain microvasculature is the blood–brain barrier (BBB), which controls the movement of molecules between blood circulation and the brain parenchyma. In this review, we are focusing on tight junctions (TJs), the multiprotein complexes that play an important role in establishing and maintaining barrier function. After a short introduction of the cell types that modulate barrier function via intercellular communication, we examine how age, age related pathologies and the aging of the immune system affects TJs. Then, we review how the TJs are affected in age associated neurodegenerative disorders: Alzheimer’s disease and Parkinson’s disease. Lastly, we summarize the TJ aspects of Huntington’s disease and schizophrenia. Barrier dysfunction appears to be a common denominator in neurological disorders, warranting detailed research into the molecular mechanisms behind it. Learning the commonalities and differences in the pathomechanism of the BBB injury in different neurological disorders will predictably lead to development of new therapeutics that improve our life as we age.

## 1. Introduction

Aging and age-related co-morbidities are rapidly increasing unresolved health and socio-economic problems in developed countries. Decline of cognitive brain functions represents one of the main health challenges of aging and includes vascular and neurodegenerative dementias such as Alzheimer’s or Parkinson’s disease.

The functional state of the central nervous system (CNS) is greatly dependent on the quality of the vasculature. As the centuries old saying goes: “A man is as old as his arteries”. Today, especially for the brain, this concept should be redefined: You are as old as your microvessels and capillaries [1]. There is increasing evidence that the cerebral microvasculature and the neurovascular unit play a critical role in age-related brain dysfunctions. The multitude of brain microvascular changes accompanied by aging includes endothelial dysfunction, blood–brain barrier (BBB) breakdown, decrease in blood flow, microhemorrhages, vessel rarefication and neurovascular uncoupling. In this review, our main focus is the breakdown of the paracellular barrier and tight junctions.

## 2. Cells of the Neurovascular Unit (NVU)

The vasculature in the brain forms a functional unit with the surrounding neural tissue, thus the term neurovascular unit was coined [2]. A functionally intact neurovascular unit (NVU) is a prerequisite for the proper function of the CNS. The most important cellular components of the NVU are cerebral endothelial cells, pericytes, astrocytic endfeet and neurons; however, other cellular elements like microglia may also play a modulatory role. The main role of the NVU besides neurovascular coupling is the formation of the BBB.

Cerebral endothelial cells (CECs) lining brain capillaries are considered the principal barrier forming endothelial cells. They are interconnected by a continuous line of tight junctions and characterized by a high number of mitochondria and low number of caveolae [3,4,5]. These characteristics contribute to the formation of a paracellular and transcellular barrier.

Pericytes are localized in the duplication of the basement membrane covering the basal surface of the endothelium. The estimates of pericyte coverage show large variations in the literature [6,7]. Pericytes can secrete a large number of substances that may influence endothelial function including TGFβ, angiopoetin-1 or VEGF. It seems that the differentiation stage of pericytes determines their effect on the endothelium as well [8]. The role of pericytes in the formation of the BBB is supported by the finding that absence of pericytes leads to endothelial hyperplasia, abnormal vasculogenesis and an increased BBB permeability [9,10].

Although the role of astrocytes in the formation of the physical barrier is limited, due to their influence on cerebral endothelial cells they play an important role in the maintenance of the BBB [11]. The astrocytic endfeet ensheath the brain vasculature almost completely [12] and express the water channel protein, aquaporin 4, which is suggested to play a crucial role in creating a bulk flow in the brain parenchyma from arterioles towards venules. This flow was shown to contribute to the clearance of extracellular proteins and metabolic waste products through the newly rediscovered glymphatic system [13,14]. Despite much interest in the glymphatic system, some experimental results do not support or even contradict its function [15]. Thus further refining of the glymphatic hypothesis is likely necessary [16]. Nevertheless, the aquaporin 4 in astrocytic endfeet is indispensable to BBB function as its knockout results in altered brain microvasculature and decreased water exchange through the BBB [17], furthermore its subcellular distribution shows an age dependent depolarization accompanied by decreased protein clearance [18].

The BBB is in the forefront of the defense line of the CNS and restricts the free movement of solutes and cellular elements between the systemic circulation and neuronal tissue. The BBB is involved in the pathogenesis of a large number of CNS disorders [19].

Cell types comprising the NVU are in close communication in order to maintain physiologic function and react to pathologies. Ligand-receptor type intercellular interactions and ion channels were described early as pathways that coordinate the function of the cell types constituting the NVU [20]. An example of bidirectional information exchange is the role of CD146 in coordinating the development of pericyte coverage on brain vasculature during early ontogenesis. At first CD146 is expressed by endothelial cells but as pericyte coverage increases, CD146 expression shifts to pericytes where it acts as a co-receptor of PDGFRβ. Endothelia attached pericytes down-regulate endothelial CD146 via TGFβ1 secretion, promoting BBB maturation [21]. Recently a growing body of evidence suggests that extracellular vesicle—mainly exosome—mediated bidirectional communication coordinates key functions of the NVU at the local and systemic level as well [22,23,24,25]. In a mouse model of spinal cord injury, pericyte-derived exosomes improved microcirculation and protected barrier function [26]. In response to traumatic brain injury, the loss of pericytes and consequent impairment of crosstalk among NVU cells causes barrier dysfunction, brain edema and leakage of cerebral vasculature. A recent example of pericyte to endothelial cell communication in the retina is the circular RNA cPWWP2A that is synthesized in pericytes and downregulates angiopoietin 1, occludin and sirtuin-1expression in endothelial cells by sequestering miRNA-579 [27].

## 3. Brain Capillaries in Aging

With aging, the density of brain vasculature is decreased and cerebrovascular dysfunction appears to precede and accompany cognitive dysfunction and neurodegeneration. Cerebrovascular angiogenesis is decreased and cerebral blood flow is inhibited by anomalous blood vessels such as tortuous arterioles and thick collagen deposits in the walls of veins and venules [28].

In most mammals, the capacity of CECs to divide is limited and endothelial cells are prone to be senescent. Aging is associated with endothelial dysfunction, arterial stiffening and remodeling, impaired angiogenesis, defective vascular repair and with an increasing prevalence of atherosclerosis [29]. In the aging brain cerebral blood flow declines and perfusion pressure either is constant or increases. In Brown-Norway and Fisher 344/Brown-Norway rats that maintain a relatively consistent cortical volume throughout life the densities of arterioles and arteriole-to-arteriole anastomoses on the cortical surface was found to be decreased with age [30]. In a spontaneously hypertensive rat model, long term hypertension was found to gradually destroy BBB, resulting in white matter lesions, one of the most important pathological changes in vascular dementia [31]. At the capillary level, increased capillary diameters and decreased capillary density paired with increased red blood cell velocities were observed [28,32]. Some capillary density measurements in humans contradict these observations, as no changes were observed in the intervascular distance on CD31 stained brain sections [33].

## 4. Junctional Proteins in Aging and Related Disorders

The paracellular barrier properties of CECs are determined by the tight junction (TJ), which are composed of transmembrane proteins that control the transport across the intercellular space between adjacent cells and cytoplasmic plaque. Claudin 5, member of the 27 strong claudin family, is primarily responsible for controlling the paracellular transport of water and small molecules through capillary vessel walls [34]. Other claudins may contribute to BBB function as well, though the isoforms present in human and murine brain vessels is still under investigation [35,36]. Like claudins, MARVEL (MAL and related proteins for vesicle trafficking and membrane link) proteins especially occludin play a role in restricting paracellular transport and also regulate homeostasis and TJ organization [37,38,39,40]. The third group of integral membrane proteins of the TJ belongs to the CTX (cortical thymocyte marker in *Xenopus*) family within the immunoglobulin superfamily such as junctional adhesion molecules (JAM) and coxsackie and adenovirus receptor (CAR). Integral membrane proteins of the TJ are coupled to cytoplasmic plaque proteins that provide a platform for anchoring the junction to the cytoskeleton and for interactions with signaling molecules. The main components of the cytoplasmic plaque are zonula occludens (ZO) proteins [41,42,43].

Junctional proteins are not restricted to the endothelial cells of the NVU. Recently, pericytic occludin was described in a new role as a NADH oxidase enzyme and an important player in BBB pericyte metabolism including the modulation of pericyte energy sharing with other NVU components via intercellular transport of mitochondria and glucose [44]. Astrocytic occludin was described two decades ago [45], and a recent report proposed a new role for it in RNA metabolism [46]. Astrocytic TJ, composed of claudin 1, 4 and JAM may also form in inflammation to control lymphocyte segregation [47].

### 4.1. TJ in Aging and Aging Models

Limited data is available on what changes develop in the function of the BBB and the composition and structure of endothelial TJs in the healthy aging human brain. In a meta-analysis of BBB permeability studies, the barrier function was negatively impacted by age. Though there were some discrepancies, paracellular permeability was generally increased in the aged human brain [48]. Multiple accelerated aging animal models are available to complement this data, which we will discuss mainly together with the appropriate aging associated disorders in later paragraphs. Literature data presented in this chapter are summarized in Table 1. In the review we will include large molecule (e.g., albumin or Evans blue albumin) permeability and blood component extravasation as well even though these are not markers of TJ dysfunction but rather indicate transcellular transport or disruption of the vascular wall respectively.

Experimental results linking BBB dysfunction to declining cognitive function prompted the use of modern in vivo imaging such as dynamic contrast enhanced magnetic resonance imaging to study cerebrovascular permeability. A recent work revealed an age dependent BBB permeability increase in the hippocampus of individuals without any cognitive impairment, which was exacerbated in the case of mild cognitive impairment of Alzheimer’s, Huntington’s and multiple sclerosis patients [58].

Permeability changes are likely the result of decreased expression and disorganized localization of TJ proteins. As shown in the BubR1 hypomorphic murine model of accelerated aging, claudin 5, occludin and ZO-1 expression of cortical and striatal microvessels were decreased in aged mice, with reactive astrogliosis also apparent at the vessels. BubR1 is a key member of the mitotic checkpoint that ensures accurate chromosome segregation [49]. In an experiment using cells isolated from BubR1 hypomorphic mice, aged CECs (co-cultured with pericytes) showed a discontinuous junctional staining for claudin 5, occludin and ZO-1 and the presence of abnormal cells with smaller cytoplasm that are highly immune-reactive for TJ proteins [59]. The BubR1 hypomorphic CECs also showed increased number of spikes in the claudin 5 staining, reminiscent to those previously described as budding and docking sites for claudin bearing vesicles [60].

Aged mouse and human brains both showed fragmented and weak claudin 5 and ZO-1 staining in microvessels compared to healthy young controls, with an accompanying reduction of claudin 5 protein expression. BBB permeability to 3 kDa dextran was also increased in aged mice. Both claudin 5 levels and BBB function were rescued in transgenic mice overexpressing sirtuin-1, a NAD-dependent deacetylase, while inducible, endothelial-specific knockdown of sirtuin-1 increased barrier permeability and reduced claudin 5 mRNA and protein levels [50]. These results add a novel mode in which sirtuin-1 affects longevity and neurological disorders by maintaining BBB integrity [61,62].

Barrier function of the cerebral vasculature was shown to be under the control of estrogen [63]. Thus, a potential influence of the age-dependent decline of estrogen levels on BBB integrity in postmenopausal women or reproductive senescent female animals appeared plausible. In this context, Bake and Sohrabji [51] showed enhanced extravasation of Evans Blue-albumin in the hippocampus and olfactory bulb of reproductive senescent female rats. Moreover, the localization of the tight junction protein claudin 5 in hippocampal microvessels was affected in senescent female rats compared to young adult ones. In case of human brain microvessels, disrupted distribution and poor junctional localization of claudin 5 was altered in postmenopausal women, in contrast to premenopausal women [52]. Further confirmation to suggest that reproductive hormones regulate BBB permeability came from studies with ovariectomized rats [53]. While expression of ZO-1 was unaffected, redistribution and increased expression of connexin-43 was observed following ovariectomy. Estrogen replacement to young (4 months old) ovariectomized rats restored BBB function, while reproductive senescent animals did not benefit from estrogen treatment or even showed increased BBB permeability in the hippocampus [53].

Further evidence of age-related junctional alterations at the BBB came from a study showing that the cerebral occludin protein content in total cerebral tissue extracts from 24-month-old rats was significantly reduced compared to 12-month-old or 3-month-old male rats without a corresponding change in occludin mRNA. ZO-1 protein levels were found to be unchanged while ZO-1 mRNA was significantly increased in 24-month-old rats compared to 3-month-olds [55].

By using transmission electron microscopy, a decline in the pericytic coverage of aged capillaries has been described over thirty years ago [64]. Beyond the assumption that the age-dependent loss of pericytes decreases the ability of the BBB to compensate for transient leaks, there is evidence that pericytes regulate the expression of TJ/BBB-specific proteins in microvascular endothelial cells. A comparison of claudin 5, ZO-1 and occludin expression in 6–8 month old Pdgfrβ+/− mice has revealed significant reductions of protein expression in pericyte-deficient mice compared to controls [54]. Therefore, it is likely that aging-associated TJ dysregulation may be partly the consequence of pericyte loss.

Integrins are heterodimeric proteins that anchor cells to the extracellular matrix and are involved in ligand dependent bidirectional signaling between the cell and its environment thus playing important roles in all cell functions. Researchers have found connections between integrins and aging [65,66] and neurological disorders [67]. Disruption of cerebral endothelial integrin interaction with the basal lamina lead to decreased claudin 5 levels and BBB integrity [56]. Recently, integrin α5 knockout mice were found resistant to transient middle cerebral artery occlusion (MCAO) induced ischemic stroke with a small initial infarct volume that disappeared within 3 days. The α5 knockout animals showed improved barrier function and higher claudin 5 mRNA levels compared to controls [57].

CTX family proteins have not been directly linked to aging; however, some play important roles in the brain development and function or tumorigenesis and invasiveness. JAM-A in CECs undergoes subcellular relocalization in response to inflammatory stimuli [68] and its levels are reduced in response to brain injury [20]. JAM3 was found to be essential for BBB integrity and for normal lens development in humans. Homozygous mutations of JAM3 in humans led to brain hemorrhage and brain calcification [69], the latter being somewhat similar to calcification in the basal ganglia in occludin knockout mice [70]. Brain endothelial function of CAR is scarcely known. However, it was found to play key roles in neurons in synapse development in the postnatal and adult brain and its loss contributes to cognitive defects [71,72].

### 4.2. Brain Endothelial TJ in Ischemic Injury

In the healthy brain, practically every neuron has a capillary running next to it. With the rarefaction of capillaries in aging brain, the ability to maintain homogeneous flow during periods of localized ischemia is reduced. Hypoxia and reoxygenation are important components of many disorders that affect the central nervous system, including stroke and dementia [73,74,75]. The incidence of stroke is increased with age and the prognosis of elderly stroke patients is unfavorable compared to young adults [76,77]. Literature data regarding TJs presented in this chapter are summarized in Table 2.

In vitro studies of hypoxia and hypoxia followed by reoxygenation have revealed much of the cellular mechanisms affected by ischemia in CECs. In oxidative stress, the interactions of occludin with claudins or proteins of the ZO family are affected, directly influencing the formation and function of the TJ. Oxidative stress downregulates occludin, reduces its specific membrane localization and regulatory contribution to barrier tightness via multiple signaling pathways [102].

Significant disruptions were seen in the distribution pattern of occludin after hypoxia [78]. These changes were less apparent in posthypoxic reoxygenation, correlating to the increased protein expression [79]. MCAO caused NADPH oxidase upregulation, ROS (reactive oxygen species) generation, matrix metalloproteinase (MMP)-9 activation, and edema formation [103]. MMP activity in oxidative stress increases paracellular permeability by occludin cleavage. However, applying the same level of MMP activity without oxidative damage, neither is occludin cleaved nor BBB permeability is increased [80]. In an opposite approach, normobaric hyperoxia protected the BBB, and the expression and distribution of occludin against MMP-9–mediated effects in cerebral ischemia [103]. Thus occludin plays a key role in the response of cellular barriers to redox changes and could be a redox sensor at the TJs [104,105]. In a brain endothelial cell culture model of hypoxia/reoxygenation, ERK1/2 was activated and the amount of occludin and barrier function decreased which was exacerbated by glucose deprivation [81]. Hypoxia (1% O_2_) altered the location of claudin 5 in the plasma membrane and the level of claudin 5 protein in bEND.3 cells, and these changes were accompanied by an increase in BBB permeability. In vivo, claudin 5 was also significantly reduced under hypoxic conditions [82]. ROS-induced BBB disruption via the redistribution and decrease of claudin 5 and occludin levels was paralleled by cytoskeleton rearrangements. This rearrangement was mediated by RhoA, PI3 kinase and protein kinase B (PKB/Akt), and specific inhibitors prevented ROS-induced monocyte migration across an in vitro model of the BBB [83].

The effect of age on the outcome of ischemic stroke was studied in murine distal MCAO experiments, demonstrating an increased inflammatory reaction in 12 month old mice compared to 2 month olds based on IL-6 and IL-1β levels in the ischemic tissue. At the same time, there was an increased number of CD68 immunoreactive cells, denoting phagocytosis and microglia activation in peri-infarct tissue and the older mice suffered more severe BBB damage as well [84]. Similar BBB permeability increase was observed in young adult rats after one hour of hypoxia (6% O_2_) followed by 10 minutes of reoxygenation, which also lead to increased occludin phosphorylation without significant changes in ZO-1 expression [85]. Disrupted occludin staining of brain endothelial cells in rats was reversible by administration of ROS scavengers [73]. Another study under similar conditions showed PKC-dependent focal BBB leakage and disrupted claudin 5, occludin and ZO-1 organization at endothelial cell borders while the protein levels were slightly increased in the membrane fraction for claudin 5 and occludin [86].

Functional changes of the BBB appear shortly after a hypoxic event, but structural TJ changes develop much slower. Using an EGFP-claudin 5 transgenic mouse model, researchers demonstrated a stepwise alteration of the BBB, leading to an increase in transcytosis followed by alterations of TJ proteins, which undergo significant ultrastructural remodeling and localization changes starting at 48–58 h post tMCAO (transient middle cerebral artery occlusion) [87]. Early permeability increase elicited by tissue plasminogen activator (tPA) during reperfusion in elderly rats was accompanied by greatly increased claudin 5 phosphorylation while unphosphorylated claudin 5 decreased compared to the contralateral side. MCAO treatment itself increased claudin 5 phosphorylation slightly. Occludin protein level was decreased in elderly compared to young rats and further degradation was observed in response to MCAO and tPA. These age-dependent differences in claudin 5 phosphorylation and occludin degradation were inferred from redistribution of western blot bands [88].

In a multifocal cerebral ischemia model, Src kinase dependent tyrosine phosphorylation of occludin was found to contribute to barrier disruption. Systemic inhibition of Src resulted in decreased infarct size, decreased VEGF-A, rescuing claudin 5, occludin expression and barrier function [89,90,91]. Reperfusion experiments following 90-minute hypoxia in spontaneously hypertensive rats revealed MMP dependence of BBB leakage and the disruption of claudin 5 and occludin at the TJ in vivo. Interestingly the TJ proteins from disrupted endothelial barrier appeared in adjacent astrocytes underlining intercellular communication [92,93]. In vivo use of the MMP inhibitor GM6001 in rats as an early treatment given at the time of MCAO increased the number of vessels and improved ZO-1, occludin and claudin 5 expression in the infarct region 3 weeks after the ischemic injury [94].

In a sterile inflammation model induced by TNF-α in human primary brain microvascular endothelial cells, PARP inhibition promoted BBB barrier function. Furthermore, PARP inhibition in primary endothelial cells improved TEER and increased occludin and claudin 5 protein levels [95]. In spontaneously hypertensive rats that underwent MCAO, minocycline, an inhibitor of PARP-1 and anti-inflammatory, anti-apoptotic and neuroprotective drug, significantly reduced the infarct size and prevented tissue loss, improved perfusion, reduced BBB permeability and increased ZO-1, occludin and claudin 5 protein levels. At the same time, increased MMP-2 and -3 were detected at four weeks, at which time peri-infarct microglia showed M2 activation [96].

In a recent genetic model of intracerebral hemorrhagic stroke and vascular dementia, cerebral small vessel disease symptoms were elicited by inducible knockdown of the transcription factor, serum response factor (SRF) or its cofactors Myocardin Related Transcription Factor (MRTF-A/-B). The microhemorrhagic phenotype observed in this model is attributed to loss of TJ components and is specific for brain tissue. The authors demonstrated mRNA level decrease of claudin 1, 3, 5 and 12, as well as ZO-2 and ZO-3, and significant loss of claudin 5 protein in isolated brain endothelial cells [97].

Exposing mice to normobaric hypoxia led to an increase in brain vascular permeability associated with diminished expression of occludin and inhibition of VEGF attenuated vascular leakage [78]. This confirms the results of studies in primary brain endothelial cells in which VEGF decreased occludin expression [98]. Astrocyte derived VEGF-A acts directly on brain microvasculature to downregulate occludin protein and mRNA in vitro and in mouse models of experimental autoimmune encephalomyelitis (EAE), inducing BBB permeability. In vivo inactivation of astrocytic VEGF-A expression or systemic use of the eNOS inhibitor cavtratin rescued BBB function [99].

### 4.3. Involvement of Brain Barriers in Aging-Associated Alterations of Immune Functions

Brain barriers are important interfaces for neuroimmune communication and any disturbance may inevitably result in CNS dysfunctions. Aging-associated alterations at the BBB and brain cerebrospinal fluid (BCSF) barrier are thus considered to be one trigger for the development of cognitive impairment(s) in the aged brain (reviewed in [106,107]). Literature data concerning TJs presented in this chapter can be found at the end of Table 2.

While it is well established that migration of immune cells from the blood into the CNS parenchyma is a process occurring in inflammatory and neurodegenerative state, accumulating evidence suggests that immune cells also infiltrate the perivascular space of non-diseased brain, though at very low levels, maintaining and contributing to the surveillance and homeostasis of the CNS (reviewed in [108,109,110]).

Less information exists concerning age-related immune cell trafficking across the BBB and BCSF, obviously due to the fact that normal aging of the brain is usually accompanied by at least low level inflammatory activity [111]. Leakage of IgG into the cerebral cortex and hippocampus of healthy aged (24 months old) but not young (3 months old) mice were reported [100]. This leakage was accompanied by a decrease in occludin and ZO-1 expression in cerebral endothelial cells but no signs of leukocyte transmigration into the CNS.

A progressive age-related enhancement of TNFα-elicited T cell infiltration in brain parenchyma and choroid plexus of mice was demonstrated by Xu et al. [112]. Further, Stichel and Luebbert [113] reported on the presence of T cells and dendritic-like cells in mouse brain parenchyma at about 12 months of age, with numbers increasing during aging. T cells from aged mice were found to exhibit increased expression of adhesion molecules, such as CD11a and CD49d, facilitating diapedesis [114] and elevated ICAM1 expression was found in brain vasculature of aged human and mouse brains [115].

The role of aging-associated T cell infiltration of the non-diseased brain is still unclear. Results from single-cell RNA-sequencing and immunofluorescence staining of the subventricular zone from young (3 month old) and old (28–29 months) mice suggest that T cells, expressing interferon-γ, were clonally expanded and markedly enriched in the subventricular zone from old mice [116]. In vitro studies have shown that T cells can inhibit the proliferation of neural stem cells in co-cultures in an interferon-γ-dependent mechanism [116]. Thus, it may be assumed that interaction of T cells with neural stem cells in neurogenic niches of old animals may contribute to the proliferative decline of neural stem cells during aging. A distinct role of immune cells was demonstrated in increasing cerebral vascular permeability [117]. Using a mouse model of CD8 T cell-mediated CNS vascular permeability it was shown that CD8 T cells are capable of initiating BBB disruption in a non-apoptotic perforin-dependent manner.

### 4.4. Alzheimer’s Disease (AD)

The blood–brain barrier contributes to the pathology and may play a causative role in the development of the most common neurodegenerative disease afflicting the elderly population: Alzheimer’s disease (AD). The involvement of the BBB happens through multiple mechanisms such as barrier disruption, transporter dysfunction and secretion of neurotoxic substances [118,119]. Amyloid-β peptide (Aβ), the main component of senile plaques in the AD influences the expression and localization of TJ proteins [120,121,122]. The extracellular matrix and TJ proteins are substrates of MMPs, whose activity is also increased in AD and after ischemic injury, which results in lower TJ proteins [123]. Data concerning TJ changes in Alzheimer’s, Parkinson’s and Huntington’s diseases, as well as schizophrenia are summarized in Table 3.

Similar to data on permeability in the aging brain, the BBB permeability data of AD patients was heterogeneous. This could partly be ascribed to difficulties in separating different types of dementia and co-morbidities confounding results. In a meta analysis by Farrall and Wardlaw some results show increased BBB permeability in AD compared to controls but the permeability increase is higher and more clear cut for vascular dementia and mixed dementias compared to AD [48]. Murine models of AD allow for more precise control over experiments and make it possible to concentrate on separate aspects of the disease; however, available results are still conflicting. A number of studies showed that the paracellular route is unaffected in murine models of AD. There was a substantially decreased occludin expression in APP/PS1 mice AD model compared to wild-type controls [143]. In some cases, when using an APP/PS1 mouse model there was an unaltered BBB permeability to ^131^I-albumin [144] and to sodium fluorescein [145]. Even though earlier work showed small localized disruption in Senescence Accelerated Mouse-Prone 8 (SAMP8), no global BBB permeability change was observed when comparing 12 and 4 months old animals [101]. Comparing 11 and 4 months old 3 × Tg-AD mice (triple transgenic mice for familial Alzheimer’s disease mutations), Bourasset et al. found no changes in the distribution of vascular space markers ^3^H-inulin and ^14^C-sucrose, but found a global reduction in cerebrovascular volume that was most significant in the hippocampus [124]. In a comparison of 18–20 month old 3×Tg-AD and wild type mice researchers also did not find changes in ^3^H-inulin and ^14^C-sucrose uptake, while the uptake of passively diffusing markers ^3^H-diazepam and ^3^H-propranolol was less in AD mice [146]. These results suggest that in murine AD models global BBB breakdown is not detectable and research probably should focus on local barrier disruption, as data from multiple research groups point to the importance of cerebral amyloid angiopathy in AD and there is ample amount of research revealing the molecular mechanisms behind it.

Regarding TJ proteins in post-mortem human brain, a dramatic loss of claudin 5, occludin and ZO-1 was demonstrated in Aβ-laden capillaries surrounded by NADPH oxidase-2 (NOX-2)-positive activated microglia. Further results show that Aβ is toxic to endothelial cells in the human brain via binding to the receptor for advanced glycation end-products (RAGE) and induction of ROS production, which ultimately leads to disruption of TJs and loss of BBB integrity [125,126]. In a model of age-related macular degeneration, increased formation of ROS was found after administration of the oligomeric form of Aβ1–42 to retinal pigmented epithelial cells [127], which was accompanied by a decrease in the expression of occludin. The Aβ1-42 peptide accumulation around brain microvessels was found to be mediated by transport via RAGE in 5XFAD mice, increasing permeability, disrupting claudin 5, ZO-1 and occludin. This MMP and Ca^2+^-calcineurin dependent BBB disruption could be prevented in bEnd.3 cells by neutralizing antibodies against RAGE [128], small interfering RNA knockdown of RAGE [129] or *Ginkgo biloba* extract EGb761 [130].

An in vitro study on Aβ1-42-treated bEnd.3 cells showed that AnnexinA1 (ANXA1), an anti-inflammatory messenger, significantly rescued the expression of claudin 5 and ZO-1 and barrier function in Aβ1-42 -treated bEnd.3 cells. Aβ1–42 reduced ANXA1 bEnd.3 cells, and also had reduced expression in capillaries of 5XFAD mice, and the human serum of patients with AD. ANXA1 acted via the inhibition of RhoA-ROCK signaling. In co-culture experiments, pericyte secreted ANXA1 attenuated the Aβ1–42-induced disruption of the tight junction [131].

Pericytes influence the BBB by promoting TJ protein expression in endothelial cells [54] and helping the alignment of TJs [147]. It has also been shown that a loss of pericytes plays a role in AD development and is followed by a decreased expression of TJ proteins [148,149].

The strongest genetic risk factor for late onset AD is Apolipoprotein E4 (ApoE4). ApoE4 and its receptors are expressed throughout the NVU and are linked to many aspects of cerebrovascular dysfunction [150,151]. The molecular mechanisms of ApoE4 mediated neurovascular injury demonstrate how the coordinated effort of multiple cell types maintains a functional NVU. Bell et al. revealed using multiple transgenic mice that the ApoE induced degradation of TJ proteins claudin 5, ZO-1 and occludin and basal membrane protein collagen IV is the result of an intercellular communication error. ApoE4 secreted by astrocytes is unable to bind LRP1 on pericytes and thus does not block the pericytic cyclophilin A/NF-κB/MMP9 pathway, which results in vascular dysfunction [152].

Soluble Aβ is also known to induce secretion of proinflammatory cytokines (TNF and IL-6) and chemokines, which stimulate the production of MMP-2 and MMP-9 and it also activates the production of ROS [153]. Furthermore, experimental data confirms the involvement of all cells of the NVU in the effect of Aβ. Microglia activated by Aβ treatment shows reduced expression of trophic factors that are responsible for inflammatory resolution and increased pro inflammatory NO and TNFα release. This affects both astrocytes and capillary endothelium leading to reduced BBB integrity and function [132].

AD is also accompanied by tau protein accumulation and hyperphosphorylation, which was shown to promote BBB dysfunction in AD and other tauopathies [154,155,156].

### 4.5. Parkinson’s Disease (PD)

The BBB is also involved in the progression of the second most common neurodegenerative disorder: Parkinson’s disease (PD). The contribution of BBB disruption to PD is not widely studied despite the implication of known BBB damaging mechanisms and agents such as oxidative stress and MMPs in the pathomechanism of the disease [157,158]. Thickened basement membrane in the cingulate cortex and degeneration of the brain microvasculature in PD was reported by Farkas et al. and Guan et al. [159,160]. Gray and Woulfe published the first report of BBB disruption evidenced by blood extravasation in striatal PD tissue in 2015 [139]. Motor function and regional blood flow can be improved in PD patients by deep brain stimulation [161], which is possibly a result of normalizing aberrant microvasculature in PD. In a study regarding the effects of deep brain stimulation of the subthalamic nucleus in PD patients, the decreased immunofluorescence signal of claudin 5, occludin and ZO-1 of PD samples could be rescued by deep brain stimulation treatments [138]. This is in line with multiple studies using experimental parkinsonism models. Significant decrease was detected in the amount of occludin in the striatum, which was associated with increased BBB leakage, in the 1-methyl-4-phenyl-1,2,3,6-tetrahydropyridine (MPTP)-treated mouse model. Increased striatal MMP-9 activity was also detected in the MPTP model with a possible role in TJ opening [133,134]. Increased permeability of the BBB was also observed in the striatum in rats after treatment with unilateral injections with 6-Hydroxydopamine (6OHDA) [135] that leads to downregulation of occludin [136]. Similar to amyloid oligomers in AD the Lewy body component α-synuclein was found to damage BBB. An in vitro study showed that preformed α-synuclein fibrils lead to a decrease in the expression of occludin and ZO-1, but not tricellulin or marveld3, in human brain microvascular endothelial cells [137].

### 4.6. Huntington’s Disease (HD)

Huntington’s disease (HD) is a dominantly inherited autosomal neurodegenerative disorder that is not usually considered aging related as it is typically diagnosed at the age of 40, and the onset of the disease can vary from earlier than ten to over eighty years of age. However, recent research indicates that epigenetic age acceleration in specific brain regions is associated with HD [162].

Recently, morphological changes of blood vessels and BBB leakage in the caudate and putamen were observed in HD patients using magnetic resonance imaging. In putamen samples of HD patients and in striatal samples of the HD model R6/2 mice, occludin and claudin 5 protein levels were decreased and evidence of increased BBB permeability was found [140]. More recently, these results were expanded in induced pluripotent stemm cell (iPSC) derived brain microvascular endothelial cells. While in this system no significant changes in TJ protein levels were observed, claudin 5 was found to mislocalize to the cytoplasm of cells derived from HD patients; however, trans endothelial electric resistance (TEER) and MDR1 function was significantly decreased compared to control cell lines. Some of the changes in cell lines derived from HD patients could be attributed to aberrant Wnt signaling [141].

### 4.7. Schizophrenia

Some aspects of schizophrenia raise the possibility that accelerated aging of the brain can be responsible for some of its symptoms [163]. Individuals with the chromosomal abnormality: 22q11 deletion syndrome (22q11DS) have an increased risk of developing schizophrenia, in fact it occurs in 30% of such individuals. Interestingly, 22q11DS are haploinsufficient for claudin 5. Recent experiments on AAV mediated claudin 5 suppression in mice and inducible claudin 5 knockdown uncovered focal BBB breakdown and behavioral changes. Anti-psychotic medications were found to dose dependently increase claudin 5 expression. Furthermore post mortem analysis of schizophrenic brains revealed aberrant, discontinuous expression of claudin 5 [142].

## 5. Closing Remarks

Aging comes with the deterioration of all bodily functions of which the loss of cognitive function is possibly the most serious in our heavily information dependent society. With the building evidence that dysfunction of the microvasculature is not just coincident but is part of the underlying mechanisms of aging and associated neurovascular and neurological disorders, new therapeutic possibilities are opened. The significant heterogeneity of BBB disruption data in studies using aging postmortem brain tissue suggests that more data is necessary to clearly understand the role of BBB disruption and to see whether it is a symptom or a cause [48]. It is not surprising that human BBB data before the mid-1990s is mainly regarding permeability; however, even since the discovery of occludin, zonula occludens proteins and claudins, BBB TJ data in the aging brain is scarce and this deficit is just partly made up for by data from aging associated disorders. Furthermore many studies disregard that claudin 5, occludin or ZO1 does not constitute the paracellular barrier by itself and all three should be studied along with permeability to different molecular size tracers at the same time to get a picture of barrier status. Worsening the situation is that the reliability of existing TJ data has been questioned recently [35]. Thus further comprehensive BBB TJ and permeability studies are needed in the field of aging and aging associated disorders. Another focus needs to be the study of both classical and novel intercellular communication pathways between brain capillary endothelium, pericytes, astrocytes, microglia and neurons as the concerted activity of all these cell types is necessary for proper neural function. We are just beginning to understand the depth and mechanisms of intercellular information exchange that makes the NVU a functional unit. The knowledge of how the interdependent functions of the cell types constituting the NVU are affected by the process of aging can lead to the alleviation of age related impairments and a better quality of life.

## Figures and Tables

**Table 1:** Blood–brain barrier (BBB) function and junctional proteins in aging.

Experimental System	Claudin 5	Occludin	ZO-1	Permeability	Other	Authors
BubR1 accelerated aging, cell culture	Discontinuous immunofluorescence for all three.					Baker et al., 2011 [49]
aged mouse and human brain tissue	fragmented, weak immunofluorescence, reduced protein level.		fragmented, weak immunofluorescence	increased 3kDa dextran permeability rescued by sirtuin-1 overexpression.		Stamatovic et al., 2019 [50]
sirtuin-1 overexpression	rescued by sirtuin-1 overexpression					
sirtuin-1 knockdown	reduced claudin 5 mRNA and protein			increased		
senescent female rats				Evans blue extravasation, hippocampus, olfactory bulb		Bake and Sohrabji 2004 [51]
senescent rats	weaker and discontinuous immunofluorescence in hippocampal vessels in senescent compared to young female rats. No change in middle aged vs. young male rats	no change in reproductive senescent vs. young female rats			age and gender dependent IgG extravasation, e.g., increased IgG extravasation in senescent female rat thalamus	Bake et al., 2009 [52]
postmenopausal women	altered distribution					
ovariectomized rats			unaffected	increased	connexin43 redistribution	Wilson et al., 2008 [53]
+estrogen replacement				rescued	rescued	
Pdgfrβ+/−, pericyte deficient mice	All three decreased.					Bell et al., 2010 [54]
24-month-old rats, compared to 12-months-old		protein decreased, mRNA unaffected	mRNA increased, protein unaffected			Mooradian et al., 2003 [55]
24-month-old rats, compared to 3-months-old		protein decreased, mRNA unaffected	protein unaffected			
mouse primary brain microvessel endothelial culture. disrupted endothelial integrin/basal lamina interaction	decreased			increased		Osada et al., 2011 [56]
integrin α5 knockout mice	increased mRNA			improved barrier	resistant to MCAO induced stroke	Roberts et al., 2017 [57]

**Table 2:** BBB function and junctional proteins in age related disorders, ischemic injury and immune system.

Experimental System	Claudin 5	Occludin	ZO-1	Permeability	Other	Authors
hypoxia, 8% O_2_, mouse, brain lysate	unaffected	decreased protein levels	unchanged protein levels	Na-fluorescein leakage increased	MMP and VEGF dependence	Bauer et al., 2010 [78]
		disrupted immunofluorescence for both				
hypoxia in primary bovine brain microvessel endothelial culture		membrane localization perturbed	ZO2 membrane localization perturbed, ZO1 and ZO2 diffuse cytosolic staining	sucrose permeability increased	actin stress fibers, increased actin protein	Mark and Davis 2002 [79]
H_2_O_2_ ROS	unaffected	phosphorylation change, degradation			TEER increase	Lischper et al., 2010 [80]
+GM6001		rescues degradation			does not rescue	
hypoxia/reoxygenation modeled by DMNQ treatment of primary mouse brain microvessel endothelial culture		protein decreased				Krizbai et al., 2005 [81]
hypoxia in bEND.3	altered localization, decreased protein			increased		Koto et al., 2007 [82]
reactive oxygen species	altered localization, decreased protein for both				xanthine oxidase and 100 μM hypoxanthine	Schreibelt et al., 2007. [83]
MCAO, 12 months vs. 2 months old mice	decrease in both age groups, after 3 days less protein in old compared to young		decrease in both age groups, after 1 and 3 days less protein in old	larger Evans blue extravasation in old with stroke		Shen et al., 2019 [84]
young adult rats hypoxia/reoxygenation		phosphorylation	no change in protein	increased		Witt et al., 2003 [85]
young adult rats hypoxia/reoxygenation		disrupted				Lochhead et al., 2010 [73]
as above + free radical scavenger tempol		rescued				
young adult rats hypoxia/reoxygenation	disrupted localization, slightly increase membrane fraction	disrupted localization, slightly increase membrane fraction	disrupted localization	PKC-dependent focal BBB leakage		Willis et al., 2010 [86]
EGFP-Claudin 5 transgenic mouse tMCAO	eGFP-claudin 5 no change	no change	no change		Claudin 5 transgene protein expression is double of wild type.	Knowland et al., 2014 [87]
after 24–30 hours						
after 48–58 hours	eGFP-claudin 5 non membrane	reduced	reduced			
600–950 g rats compared to 200–250 g rats		degradation				Kaur et al., 2011 [88]
MCAO, both ages	slightly increased phosphorylation					
tPA after reperfusion, only in old	greatly increased phosphorylation	increased degradation				
rat tMCAO +PP2 Src kinase inhibitor, brain tissue	time dependent decrease of claudin 5 protein and mRNA was rescued by PP2			brain lysate protein decrease was significant from 24, mRNA from 72 hours and persisted 7 days later, fibrinogen leakage from brain coincided claudin 5 decrease and vas rescued by PP2		Bai et al., 2014, [89]
rat microsphere embolism, capillary lysate		decreased amount, increased phosphorylation 6–72 h after microsphere injection	decreased amount	albumin leakage starting from 48 hours		Kago et al., 2006 [90]
rat microsphere embolism, capillary lysate, +PP2		decreased amount and increased phosphorylation rescued by PP2		albumin leakage 6-24h, rescued by PP2		Takenaga et al., 2009 [91]
spontaneously hypertensive rats tMCAO, brain sections	rapid reorganization in localization 3 h after reperfusion + MMP2 activity at vessels. Protein appeared in astrocytes.			Increased gelatinase activity 3 h after reperfusion, rescued by MMP2 inhibition.		Yang et al., 2007, Yang and Rosenberg 2011 [92,93]
brain lysate	decreased mRNA, degraded protein rescued by BB-1101 MMP inhibitor					
spontaneously hypertensive rats tMCAO+GM6001 MMP inhibitor	protein levels were rescued for all three					Yang et al., 2013 [94]
Primary human brain microvascular endothelial cells+PARP inhibition	increased protein levels for both					Rom et al., 2015 [95]
spontaneously hypertensive rats tMCAO + mynocicline	increased protein levels for all three					Yang et al., 2015 [96]
SRF, MRTF-A/-B knockdown hemorrhagic stroke model, whole brain tissue	claudin1, 3, 5 and 12 mRNA and claudin5 protein downregulated		ZO-2 and ZO-3 mRNA downregulated, ZO-1 unaffected			Weinl et al., 2015 [97]
bovine brain microvessel endothelial cells +VEGF		distribution changed from membrane to cytoplasm, protein levels increased		permeability increase, resistance decrease		Wang et al., 2001 [98]
adenoviral expression of IL-1 in VEGFA knockout mouse	VEGFA KO rescued protein expression of brain microvessels in inflammatory lesions for both					Argaw et al., 2012 [99]
EAE in VEGFA KO mouse	VEGFA KO rescued protein expression of brain microvessels in inflammatory lesions					
VEGFR2 blocking antibodies in Cortical VEGF injected wild type mice	rescued protein expression for both					
eNOS inhibition or silencing in human brain microvessel endothelial cells	rescued protein expression					
24 months old mice compared to 3 months old, via fluorescence activated cell sorting	significantly decreased		decreased	IgG leakage	neurovascular inflammation and neuronal stress	Elahy et al., 2015 [100]
SAMP8 mice, 12 vs. 4 months old, permeability				no change in albumin permeability and insulin uptake		Banks et al., 2000 [101]

**Table 3:** BBB function and junctional proteins in age related disorders, neurodegenerative diseases.

Experimental System	Claudin 5	Occludin	ZO-1		Permeability	Other	Authors
Triple-transgenic mice (3 × Tg-AD), 10–11 vs. 4 month old					no change in [3H]-inulin and [14C]-sucrose uptake	Age dependent reduction in cerebrovascular volume, thickened basal membrane	Bourasset et al., 2009, Mehta et al., 2013 [124]
18–20 month old 3 × Tg-AD vs. wild type mice					no change in [3H]-inulin and [14C]-sucrose uptake decreased uptake of passively diffusing markers: [3H] diazepam and [3H] propranolol in AD mice	thickened basal membrane	
rat brain microvessel endothelial cells +Aβ1-42 for 24–72 h, immunofluorescence	disrupted localization after 24 h, increased fluorescence of Claudin 5 and 1.	no change in localization. Decreased fluorescence at 24 hours.		ZO-2 diffuse localization. ZO-2 increased fluorescence at 24 h and decreased at 72 h.			Marco and Skaper 2006 [120]
capillary cerebral amyloid angiopathy (capCAA) patient brain sections	loss of protein in capillaries with amyloid β deposits						Carrano et al., 2011, 2012 [125,126]
hCMEC/D3 cell culture + Aβ1-42 for 24 h		dose dependent, ROS mediated decrease of mRNA for both					
ARPE-19 cell culture + oligomeric Aβ1-42		disrupted membrane staining, significantly decreased mRNA			increased 40 kDa FITC-dextran permeability		Bruban et al., 2009 [127]
subretinal Aβ1-42 injection in C57BL/6 J mice		Similarly disrupted membrane staining of both in young, adult and aged mice.					
bEnd.3+Aβ1-42	decreased protein level			decreased protein levels rescued by inhibition of either RAGE, calcineurin or MMP	increased 40 kDa FITC-dextran permeability rescued by inhibition of calcineurin or MMP		Kook et al., 2012 [128]
8 months old transgenic mice with five familial AD mutations (5XFAD) compared to wild type	TJ cross section length significantly decreased, by transmission electronmicroscopy					RAGE expression was increased in brain capillaries	
bEnd.3	Oligomeric, but not monomeric or fibrillar Aβ1-42 dose dependently decreased protein levels. Protein levels were rescued by silencing RAGE with siRNA or blocking RAGE with a polyclonal antibody.						Wan et al., 2015 [129]
bEnd.3 + oligomeric Aβ1–42	decreased protein levels and increased permeability were rescued by EGb761 in a dose dependent manner					Aβ1–42-Oligo induced expression of MMP-2 and MMP-9 was rescued by blocking RAGE via antibody.	Wan, Cao, et al., 2014 [130]
bEnd.3 cells+ Aβ1-42 and isolated capillaries from 5XFAD mice	exogenous ANXA1 rescued Aβ1-42 induced decrease in protein levels by blocking Rho-ROCK signaling			exogenous ANXA1 rescued Aβ1-42 induced decrease in protein levels by blocking Rho-ROCK signaling			Park et al., 2017 [131]
APOE4 transgene expressing Apoe-/- mice	protein levels were decreased dependent on the pericytic cyclophilin A/NF-κB/MMP9 dependent pathway						Bell et al., 2010 [54]
bEnd.3, CD-1 or PARP-1 KO mouse primary glial culture +Aβ1–42		endothelial protein expression reduced by activated microglia was rescued by PARP inhibition.					Mehrabadi et al., 2017 [132]
MPTP mouse model +caffeine		MPTP treated animals showed decreased protein levels, which was rescued by caffeine.			Caffeine rescued MPTP induced Evans blue leakage in striatum.	Caffeine blocked MPTP-induced increases in MMP9 activity	Chen et al., 2008 [133]
MPTP mouse model +trehalose		immunofluorescent signal was greatly diminished in MPTP animals, which was rescued by trehalose.					Sarkar et al., 2014 [134]
unilateral striatal 6OHDA injection in rats					FITC-albumin or horseradish peroxidase leakage in striatum and substantia nigra in response to 6OHDA		Carvey et al., 2005 [135]
unilateral striatal 6OHDA injection in rats	immunohistochemistry signal decreased	protein levels decreased for both					Huang et al., 2016 [136]
hCMEC/D3 (co-cultured with mouse primary astrocytes) + preformed α-synuclein fibrils		decreased protein levels					Kuan et al., 2016 [137]
Parkinson’s Disease patient tissue sections compared to age matched and deep brain stimulated patient tissue	decreased immunofluorescent staining compared to control, rescued by deep brain stimulation.				IgG extravasation		Pienaar et al., 2015 [138]
Parkinson’s disease patient tissue sections					blood extravasation		Gray and Woulfe 2015 [139]
Huntington’s disease patient putamen samples compared to control	decreased protein levels				extravascular fibrin deposition., increased leakage in caudate and putamen by DCE MRI	abnormal vessel morphology	Drouin-Ouellet et al., 2015 [140]
and R6/2 mouse model, striatal samples	decreased protein levels				increased pinocytic activity and increased albumin extravasation	morphological changes in vessel walls	
iPSC derived brain microvascular endothelial cells from HD patients	miss-localization to the cytoplasm				decreased TEER		Lim et al., 2017 [141]
schizophrenic patient brain tissue	discontinuous localization				anti-psychotic medications dose dependently increased claudin 5 expression		Greene et al., 2018. [142]

## Abbreviations

BBB	blood–brain barrier
TJ	tight-junction
CNS	central nervous system
NVU	neurovascular unit
CEC	Cerebral endothelial cells
ZO-1	zonula occludens 1 protein
MCAO	middle cerebral artery occlusion
Aβ	amyloid-beta protein
Aβ1-42	amyloid-beta peptide containing amino acids 1-42
AD	Alzheimer’s disease
MMP	matrix metalloproteinase
PD	Parkinson’s disease
HD	Huntington’s disease
